# Operating under pressure: a scoping review of the effects of climate change on general surgery

**DOI:** 10.1007/s00464-026-13407-y

**Published:** 2026-10-16

**Authors:** Kimberly P. Woo, Ali Alipouriani, Jenny H. Chang, William C. Bennett, Abby Gross, Divya Deverakonda, Nikhila Yerabandi, Mary Schleicher, Alberto Arezzo, Marina Yiasemidou, Maggie Hodges, Vittoria Bellato, Myrthe M. M. Eussen, Patricia Sylla, Shaneeta M. Johnson, Ilyssa O. Gordon, Sofya Asfaw, Benjamin T. Miller, SAGES and EAES Sustainability in Surgery Committee

**Affiliations:** 1 Department of General Surgery, Cleveland Clinic, Cleveland Clinic Lerner College of Medicine, Cleveland, OH USA; 2 School of Medicine, Case Western Reserve University, Cleveland, OH USA; 3 Cleveland Clinic Floyd D. Loop Alumni Library, Cleveland, OH USA; 4 Division of Anatomic Pathology, Cleveland Clinic, Cleveland, OH USA; 5 Department of Surgical Sciences, University of Torino, Turin, Italy; 6 Colorectal Surgery, The Royal London Hospital, Barts Health NHS Trust, London, UK; 7 Department of Surgery, School of Medicine, University of North Carolina at Chapel Hill, Chapel Hill, USA; 8 Department of Gastroenterology, Radboud University Medical Centre, Nijmegen, Netherlands; 9 Department of Surgery, Maastricht University Medical Centre, Maastricht, The Netherlands; 10 Division of Colon and Rectal Surgery, Mount Sinai Hospital, New York, NY USA; 11 Department of Surgery, Meharry Medical College, Nashville, TN USA; 12 Meharry School of Global Health, Meharry Medical College, Nashville, TN USA

**Keywords:** Surgical sustainability, Scoping review, Climate change, Temperature anomolies, Extreme weather events

## Abstract

**Introduction:**

Climate change adversely impacts human health; however, its effect on surgical diseases remains poorly understood. We conducted a scoping review to elucidate the current understanding of climate change’s role in general surgical care and care delivery.

**Methods:**

A comprehensive search of Medline, Embase, Cochrane, and CINAHL was supplemented by manual searches. Broad terms for climate change in general surgery and its subspecialties were used. Climate-related effects were defined as daily or seasonal temperature anomalies and extreme weather events (e.g., hurricanes, floods, wildfires). Full-text articles were reviewed for relevance to treatment of surgical diseases, postoperative outcomes, and access to surgical care attributable to climate change. Thematic analysis identified recurring themes among included studies.

**Results:**

Of 5110 studies, 28 met inclusion criteria. Three themes were identified: access to surgical care, shifts in surgical disease burden, and postoperative outcomes. Five studies described decreased access to care following extreme weather events, including one reporting increased breast cancer mortality after Hurricane Katrina. Eleven studies highlighted shifts in surgical disease patterns. Temperature anomalies correlated with higher rates of traumatic injuries, skin and soft tissue infections, and complicated appendicitis. Extreme weather events were associated with increased surgical trauma and emergency case volume. Five articles found temperature anomalies associated with higher postoperative surgical site infection rates, while two did not. Four articles examined outcomes after extreme weather events, including one associating wildfire smoke exposure with worse overall survival after lung resection and another identifying longer hospital stays in wildfire-exposed facilities.

**Conclusion:**

High-level evidence regarding climate change’s impact on general surgery remains limited. Emerging evidence suggests that extreme weather events and temperature fluctuations may increase demand for surgical services, disrupt timely access to treatment, and worsen postoperative outcomes. These effects may further strain healthcare systems and resources, requiring targeted mitigation and future research in this important evolving field.

Climate change is defined as long-term, persistent shifts in global average temperatures and weather patterns. While some degree of climate change may be naturally occurring, most of the changes since the 20th century have resulted from anthropogenic greenhouse gas (GHG) emissions [1, 2]. Healthcare is a significant contributor to climate change and is estimated to be responsible for roughly 8.5% of GHG emissions in the United States [3, 4]. In turn, climate change has been shown to have serious implications for human health [5]. Increasing temperatures, worsening air quality, more extreme weather events, and rising sea levels have been shown to directly or indirectly result in the exacerbation of existing medical conditions, redistribution of vector-borne disease burdens, food insecurity and undernutrition, and increase the prevalence of certain cancers [6].

Surgical practice stands among the greatest utilizers of resources and energy within healthcare. Operating rooms (OR) use up to six times more energy per square foot than other areas of the hospital, consume more than half of hospital supplies, and produce about a third of hospital waste [6–8]. Surgery alone is estimated to produce 9.7 million tons of CO2 equivalent (CO2e) emissions annually in the US, UK, and Canada [6], equivalent to those emitted by the city of Phoenix in 2016 [9]. While the contribution of surgical practice to climate change can be quantified via GHG emissions or waste production, the converse is more difficult to capture in quantitative terms. Climate change is known to impose adverse health consequences through a myriad of direct and indirect pathways. There exists no shortage of possible mechanisms by which climate change could conceptually impact surgical disease prevalence (extreme weather leading to increases in trauma or burn injuries) or surgical complications (increased postoperative cardiovascular or pulmonary complications).

Formal considerations of these relationships regarding surgery at-large are present within the literature [10]; however, more work is necessary to better understand the impact on specific surgical disciplines. Attempts to identify and characterize such a breadth of processes are complicated by the heterogeneity of diseases, patient populations, and surgical procedures spanning disparate surgical specialties. We sought to contribute to the effort to characterize the impact of climate on surgical disciplines by identifying, organizing, and summarizing literature specific to general surgery. An estimated 15–27% of global surgical operations fall within the confines of general surgery [11]. As an initial focus for this effort, the volume and breadth of general surgical practice offered a compromise between the need to study an individual specialty while retaining sufficient generalizability to inform future research for a variety of surgical disciplines. Despite these plausible mechanisms, there has been no synthesis focused specifically on general surgery, one of the largest, most resource-intensive specialties globally. This scoping review aims to systematically map existing literature, identify knowledge gaps, and define research priorities at the interface of climate change and general surgery.

## Methods

### Search strategy

For this scoping review, we followed the published Preferred Reporting Items for Systematic Reviews and Meta-analyses (PRISMA) reporting guideline extension for scoping reviews (PRISMA-ScR) [12]. A broad search strategy was created in consultation with a medical librarian (MS). The following four databases were searched on August 29, 2025: Ovid Medline® ALL, Ovid Embase, CINAHL Plus with Full Text (EBSCOhost) and Cochrane via Wiley. No filters or limiters were imposed on the search.

Keywords included “climate change,” broadly defined as changes in temperature, global warming, and extreme weather events. Not all occurrences of natural disasters can be exclusively attributed to climate change; however, specific instances of extreme weather events, such as hurricanes, flooding, landslides/mudslides, typhoons, and droughts, may exhibit correlations with, exacerbation by, or heightened frequency attributable to the effects of climate change. To maintain the focus on climate-related variables, air pollution and natural disaster events such as earthquakes were deliberately excluded from the scoping review. To capture studies related to general surgery, care terms such as “surgery,” “trauma,” “operative,” “cancer” were used. In order to capture surgical outcomes, disease, and access terms such as “postoperative” “environmental justice” “accessibility” “disparities” “demand” and “resource” were searched. Indexing and keywords were used as well as proximity and truncation operators to increase potentially relevant results. Searches were supplemented with hand search of key citations and expert suggestions. The complete search strategies are included as supplemental material.

### Inclusion and exclusion criteria

Regarding the conceptualization of this review, peer-reviewed full manuscripts written in English involving human participants were included. Gray literature, review articles, abstracts, limited case series or surgical case reports (< 20 patients), and perspective/opinion pieces were excluded. Literature on general surgery and its subspecialties such as surgical oncology, acute care surgery, trauma, and burn surgery were included as the population of focus. Non-general surgery surgical subspecialties (neurosurgery, ophthalmology, orthopedic surgery, and urology) were considered outside the scope of the review and therefore excluded.

### Data screening, extraction, and synthesis

A total of 6678 citations were identified and uploaded into Covidence, the online platform used to manage the screening and extraction process. Of these, 1520 duplicates were removed by Covidence systematic review software, (Veritas Health Innovation, Melbourne, Australia), and another 49 were manually identified as duplicates and removed. Four reviewers (JHC, KW, AA, DD) independently assessed titles, abstracts, and full text for inclusion as described above. Any disagreements were resolved by discussion or reference to an additional team member (AG or SM, AA, DD). Data extraction on publication and study characteristics included title, author(s), year of publication, journal of publication, digital object identifier (DOI), type of study design (observational, case–control, retrospective cohort, prospective cohort), year(s) of study, data source if applicable, exposure evaluated, outcome measured, and key findings. Thematic analysis was then conducted to identify recurring themes in the literature meeting inclusion criteria. Quantitative analysis of combined results of various studies was deferred due to heterogeneity in study objectives and outcomes.

## Results

**Fig. 1:**
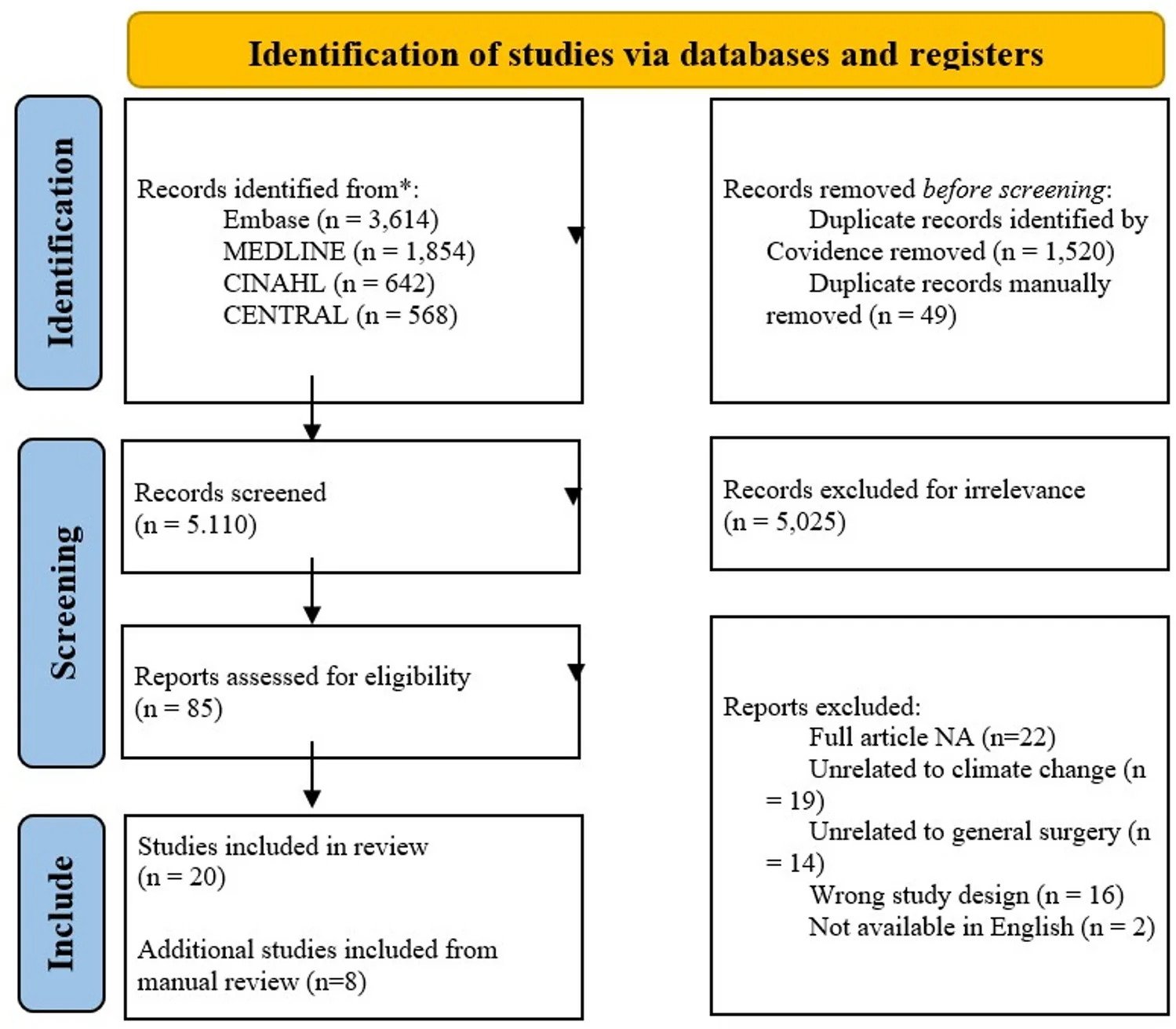
Flow diagram of study selection. Modified from: Page MJ, McKenzie JE, Bossuyt PM, Boutron I, Hoffmann TC, Mulrow CD, et al. The PRISMA 2020 statement: an updated guideline for reporting systematic reviews. *BMJ*. 2021;372:n71. https://doi.org/10.1136/bmj.n71

**Fig. 2:**
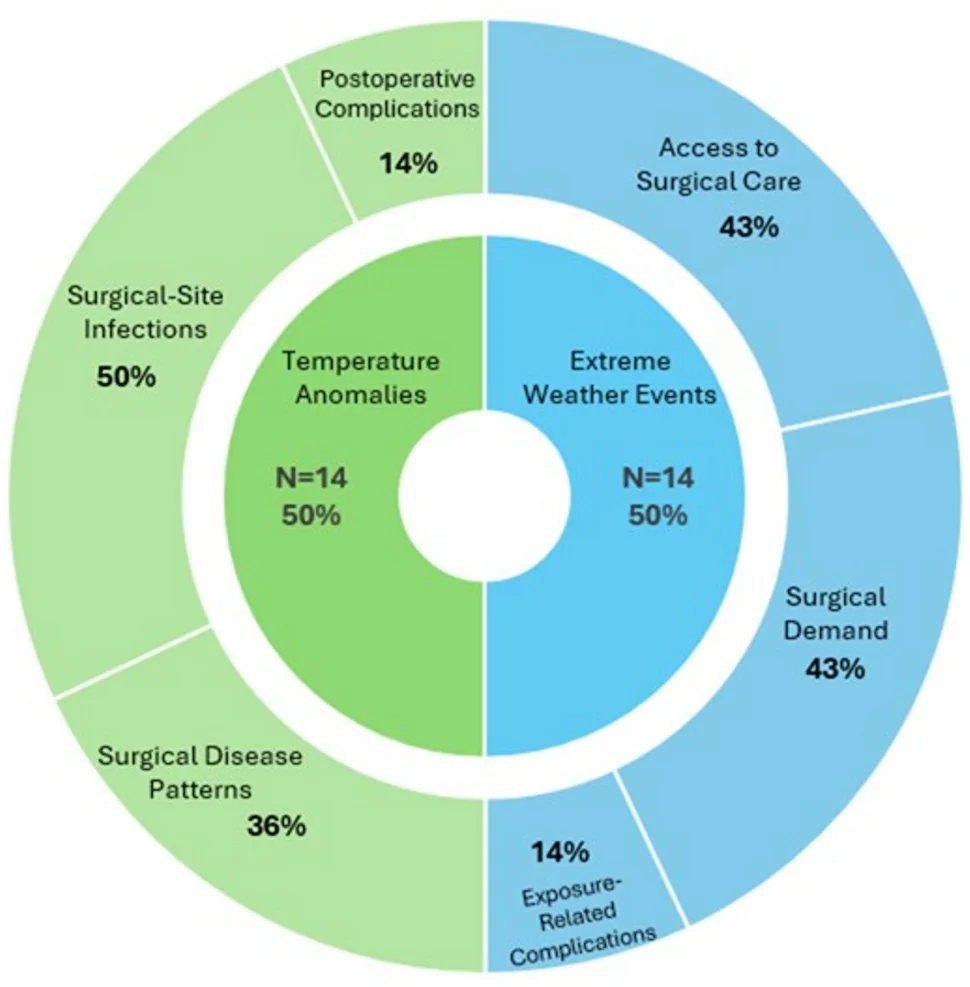
Distribution of included studies by climate change themes and subthemes

Of 5110 studies screened, a total of 28 articles were included. All studies included in our final review are described in Table 1. The process of article selection is demonstrated in Fig. 1. Methods of ascertaining climate change varied greatly among studies, ranging from deviations of daily ambient temperatures and seasonal variations to cohort studies involving exposure to extreme weather events. Two major climate change themes were identified: extreme weather events and temperature anomalies. Of the 28 articles, 14 evaluated the impact of extreme weather events (Table 1) and 14 evaluated the impact of temperature anomalies (Table 2) (Fig. 2). Across these, three recurring domains emerged: (1) increased surgical demand following climate events, (2) impaired access to surgical care, and (3) worsened perioperative outcomes and complications. Most studies were retrospective and concentrated in high-income countries, with limited representation from low- and middle-income regions despite their disproportionate vulnerability.

**Table 1:** Characteristics of included articles studying the impact of extreme weather events

Title	Authors	Year of publication	Country of study	Study design		Weather event	Outcomes
*Shifts in surgical demand*							
surgical workload of a foreign medical team after typhoon Haiyan	Read et al	2016	Philippines	Retrospective cohort		Typhoon Haiyan	Surgical workload of a field hospital
Characterizing Hospital admissions to a tertiary care hospital after typhoon Haiyan	Chang et al	2016	Philippines	Retrospective cohort		Typhoon Haiyan	Disease burden and trends in medical diseases of a local hospital and field hospital
Impact of a category-3 hurricane on the need for surgical hospital care	Sjoberg et al	2007	Grenada	Retrospective cohort		Hurricane Ivan	Surgical need of a hospital ward
Debris Flow Syndrome: Injuries and Outcomes after the Montecito Debris Flow	Langdon et al	2019	United States	Retrospective cohort		California Wildfires	Injuries from the Montecito debris flow
Mass chemical burn casualty: emergency management of 118 patients with alkali burn during a Matsa typhoon attack in Shanghai, China in 2005	Ma et al	2007	China	Retrospective cohort		Matsa Typhoon	Mass chemical burn incident
Surgical care required for populations affected by climate-related natural disasters: a global estimation	Lee et al	2016	Worldwide	Retrospective cohort		Climate-related natural disasters	Number of surgical procedures needed
*Impaired access to surgical care*							
Impact of extreme weather conditions on healthcare provision in urban Ghana	Codjoe et al	2020	Ghana	Mixed-methods		Flooding and extreme heat	Health system vulnerabilities
Effect of natural disaster on routine surgery	Breaux et al	2009	United States	Retrospective cohort		Hurricane Katrina	Logistical and financial effects on laparoscopic cholecystectomies
The Effect of Hurricane Maria on the Surgical Workload of the UPR-affiliated Hospitals	Cruz et al	2020	United States (Puerto Rico)	Retrospective cohort		Hurricane Maria	Changes in the surgical workload
Using appendiceal perforation rates to measure impact of a disaster on healthcare system effectiveness	Mack et al	2013	United States	Retrospective cohort		Hurricane Katrina	Rate of perforated appendix observed during appendectomy
Hurricane Katrina: Impact on cardiac surgery case volume and outcomes	Bakaeen et al	2008	United States	Retrospective cohort		Hurricane Katrina	30-day operative death and perioperative morbidity after cardiac surgery
The effect of exposure to disaster on cancer survival	Bell et al	2020	United States	Retrospective cohort		Hurricane Katrina	10-year survival of cancer patients
*Exposure-related complications*							
Association of wildfire exposure while recovering from lung cancer surgery with overall survival	Zhang et al	2023	United States	Retrospective cohort		Any wildfire	Postoperative long-term overall survival of lung cancer patients
Facility exposure to wildfire disasters and hospital length of stay following lung cancer surgery	Nogueira et al	2025	United States	Retrospective cohort		Wildfire disaster	Longer hospital length of stay in wildfire-exposed facilities

**Table 2:** Characteristics of included articles studying the impact of temperature anomalies

Title	Authors	Year of publication	Country of study	Study design	Exposure of interest	Outcomes
*Surgical disease patterns*						
Association of appendicitis incidence with warmer weather independent of season	Simmering et al.	2022	United States	Retrospective Cohort	Ambient temperature averaged over seven days: temperature deviations	Daily number of appendicitis cases in a given city
The meteorological influence on seasonal alterations in the course of acute appendicitis	Reinisch et al.	2017	Germany	Retrospective Cohort	Seasonal temperature; temperature deviations	Complexity of acute appendicitis presentation
Climatic factors and community—associated methicillin-resistant staphylococcus aureus skin and soft tissue infections—A time-series analysis study	Sahoo et al.	2014	India	Prospective Cohort	Ambient temperatures averaged over a week; relative humidity	Skin and soft tissue infections (SSTIs)
Effects of temperatures and heatwaves on occupational injuries in the agricultural sector in Italy	Di Blasi et al.	2023	Italy	Retrospective Cohort	Ambient temperature averaged over one day	Occupational injuries in the agricultural sector
The impact of heatwaves on workers? health and safety in Adelaide, South Australia	Xiang at el.	2014	Australia	Retrospective Cohort	Heatwaves	Work-related injuries and diseases
*Surgical site infection*						
Seasonal Impact on Surgical Site Infections in Body Contouring Surgery: A Retrospective Cohort Study of 602 Patients over a Period of 6 Years	Duscher et al.	2018	Austria	Retrospective Cohort	Seasonal temperature	SSIs
The Seasonal Variability in Surgical Site Infections and the Association with Warmer Weather: A Population-Based Investigation	Anthony et al.	2017	United States	Retrospective Cohort	Seasonal temperature	SSIs
Impact of weather on the risk of surgical site infections in a tropical area	Fortaleza et al.	2019	Brazil	Retrospective Cohort	Ambient temperatures averaged over one day; relative humidity	SSIs
Effect of climate on surgical site infections and anticipated increases in the United States	Liou et al.	2022	United States	Retrospective Cohort	Minimum and maximum daily temperatures, precipitation and specific humidity	SSIs
Increase in surgical site infections caused by gram-negative bacteria in warmer temperatures: Results from a retrospective observational study	Aghdassi et al.	2021	Germany	Retrospective Cohort	Ambient temperatures averaged over one month	SSIs
Seasonal humidity may influence Pseudomonas aeruginosa hospital-acquired infection rates	Ramos et al.	2013	Brazil	Retrospective Cohort	Temperature, relative humidity, and precipitation averaged over one month	*Pseudomonas aeruginosa* infections
Seasonal variation of common surgical site infections: does season matter?	Durkin et al.	2015	United States	Retrospective cohort	Seasonal temperature	SSIs
*Postoperative complications*						
Hot climate and perioperative outcome in elderly patients	Gautam et al.	2011	India	Prospective cohort	Peak ambient temperature	Postoperative complications
Hot climate and perioperative outcome in trauma patients	Gupta et al.	2016	India	Prospective cohort	Ambient temperature greater than or equal to 40 °C	Perioperative morbidity

### Extreme weather events

#### Shifts in surgical demand

Six studies highlighted alterations in surgical need for trauma, burn, and acute care surgery. Three of these reports described the patterns of injury following natural disasters, two of which quantitatively described the surgical workload after Typhoon Haiyan struck the Philippines in November 2013 [13, 14]. In the four weeks after impact, 70.3% of the 222 surgical procedures performed at a field hospital were for soft tissue wounds and their sequelae [13]. Another study found a statistically significant increase in the proportion of emergency surgical procedures (76.9% vs 92.2%, *p* = 0.01) during the 44-day period following impact compared to the 44 days pre-impact. This analysis also identified a shift from mostly general/gastrointestinal pre-impact (66.7%) to a majority of trauma post-impact (54.7%) [14].

Sjoberg et al. reviewed the healthcare impact of Hurricane Ivan, which struck Grenada as a category 3 Hurricane in September 2004 [15]. Compared to the same month a year prior, there is a statistically significant increase in the number of patients who were evaluated for gunshot wounds (*p* = 0.0310), diabetic foot (*p* = 0.0074), and infections from wounds (*p* = 0.0066) in the month following Hurricane Ivan. Similarly, Read et al. also found that of the 222 surgical procedures performed at the field hospital after Typhoon Haiyan, the majority (74, 33.3%) were for foot wounds in diabetic patients [13].

The secondary effects of extreme weather leading to increases in surgical demand have also previously been described. Large wildfires have become increasingly more common as a result of climate change. One study reviewed injury patterns after the Montecito debris flow, where, following large wildfires, in California, heavy rains carried mass debris down denuded hillsides [16]. The majority (92%) of patients who presented to the emergency department for injuries directly related to the debris were for soft tissue injuries, seven of whom required operative debridement and one who required eventual skin grafting. Another study described the mass alkali burn casualties after the 2005 Matsa typhoon in Shanghai, China [17]. Alkaline chemicals within a storehouse soaked by heavy rain led to 118 burn cases (45 full thickness burns), requiring 58 hospitalizations and 42 surgical procedures.

While these prior studies reviewed the impact that climate change has had on surgical disease patterns, Lee et al. performed linear regression analysis to estimate the potential surgical need for persons affected by climate-related natural disasters [18]. The authors found that between 2004 and 2014, on average, 140 million people were affected by climate-related natural disasters annually, requiring an estimated 7 million surgical procedures to care for this population. They also found there was a poor correlation between the current rate of surgical procedures performed in a given country and the estimated need for surgical care resulting from a natural disaster for that country. Countries with the least surgical capacity were found to have the greatest need for surgical care for those affected by climate-related natural disasters.

#### Impaired access to surgical care

Another result of natural disasters is impaired access to the healthcare system and surgical care. There were six studies which highlighted how extreme weather events can restrict access to healthcare and impair delivery of surgical care. Codjoe et al. explored the vulnerabilities of the health services system in Ghana during flooding and extreme heat events [19]. Flooding of roads prohibited patients from reaching health facilities. As flood waters entered hospitals, damaging equipment and supplies, healthcare activities were halted to allow for cleaning and disinfection. Extreme heat disrupted infrastructure, resulting in power outages from increased energy demands. There was also a large impact on the providers themselves, with poor ventilation and lack of cooling mechanisms, providers expressed concerns about their abilities to safely discharge their duties.

There were three studies which explored how impaired access to healthcare and surgical care may result in shifting patterns of surgical disease presentation. Breaux et al. investigated the impact of Hurricane Katrina on routine surgery, using laparoscopic cholecystectomy as an index case [20]. When comparing outcomes before and after Hurricane Katrina (allowing 3 months for infrastructure recovery), the authors found no significant difference in operative time, turnover time, or length of stay. However, compared to patients who were operated on before Hurricane Katrina, those who were operated on after were more likely to be publicly insured or uninsured, with cases post-hurricane more likely to be higher acuity and inpatient (39% vs 45%). This pattern was also observed after Hurricane Maria hit Puerto Rico in 2017 [21]. The number of surgical cases decreased by 57% in the month during and after the storm (*p* < 0.001) compared to other months within the 2-year study period surrounding the hurricane. Patients who underwent surgery after the hurricane tended to be sicker, with more patients who were American Society of Anesthesiologists physical status (ASA) class 3 or greater compared to prior to the storm (36.5% vs 29.4%, *p* < 0.001). In contrast, despite disruptions to the healthcare system infrastructure after Hurricane Katrina, Mack et al. found no significant difference in the rates of appendiceal perforation [22]. The authors used perforation of the appendix to represent progression of the disease, and therefore an indicator of access to care, concluding that there was no impact on access to emergency surgical services at this particular hospital system.

One study evaluated the impact on patient outcomes after Hurricane Katrina resulted in closure of the New Orleans Veterans Affairs Medical Center [23]. The Michael E. DeBakey Veterans Affairs Medical Center (MEDVAC; Houston, Texas) took on the extra surgical volume and more than doubled their number of cardiac operations from the year prior. They found that the patient and surgical characteristics were similar and there were no significant differences in morbidity or death compared to the year prior to the hurricane. However, natural disasters can also disrupt the continuity of care for patients with surgical diseases that require management over longer periods of time, such as cancer [24]. In unadjusted analyses, patients who were diagnosed with breast or lung cancer in the six months prior to Hurricane Katrina and were exposed to the natural disaster had an increase in mortality of 15% (HR 1.15; *p* < 0.05) compared to matched patients outside of Louisiana who were not exposed to a hurricane during the study period. Furthermore, patients diagnosed with breast cancer had significantly worse mortality (HR 1.14; 95% CI 1.00, 1.32), even when adjusting for sociodemographic characteristics and cancer stage at the time of diagnosis.

#### Exposure-related complications

Zhang et al. looked at the impact of exposure to wildfires on surgical outcomes. [25] They conducted a cohort study of patients from the National Cancer Database who underwent resection for non-small cell lung cancer (NSCLC) and mapped their surgical recovery dates to wildfire exposures. Patients who were exposed to a wildfire within 0–3 months (HR 1.43; 95% CI 1.41, 1.45), 4–6 months (HR 1.39; 95% CI 1.37, 1.41), and 7–12 months (HR 1.17; 95% CI 1.15, 1.19) after discharge from the hospital following surgery had worse overall survival than unexposed individuals. When stratified by stage (I, II, and III) at the time of diagnosis, this finding persisted, and the association was strongest in the first months after surgery for any stage.

In a complementary national analysis, Nogueira et al. evaluated postoperative outcomes among patients undergoing curative-intent lobectomy or pneumonectomy for stage I–III non-small-cell lung cancer using data from the National Cancer Database (2004–2021). Exposure was defined as the presence of a Federal Emergency Management Agency (FEMA) wildfire Presidential Disaster Declaration in the county of the treating facility between the date of surgery and hospital discharge. Patients treated during wildfire disasters had a significantly longer postoperative length of stay compared with matched unexposed patients at the same facilities (9.4 vs 7.5 days; *p* < 0.001), while in-hospital mortality did not differ [26].

### Temperature deviations

#### Surgical disease patterns

Three studies evaluated the association of deviations in ambient temperature with alterations in the presentation of two common general surgery diseases, acute appendicitis, and skin and soft tissue infections (SSTI). There were statistically significant associations between increasing incidence of appendicitis with increasing ambient temperature and higher-than-expected temperature for a particular day [27]. There was also a positive correlation between the rate of complicated (gangrenous or perforated) acute appendicitis and temperature anomolies (unusually warm or cold weather) [28]. Additionally, patients who presented after days that were one standard deviation warmer than the mean temperature had significantly higher rates of postoperative complications (18.9% vs 10.4%, *p* = 0.009). An additional study evaluated cases of SSTI found a borderline significant positive relationship with weekly average maximum temperatures above 33 °C (coef. 0.69, *p* = 0.058) [29]. There was, however, a significant positive relationship between methicillin-resistant Staphylococcus aureus (MRSA) SSTI cases and weekly average maximum temperatures above 33 °C (coef. 0.57, *p* = 0.044).

The correlation between temperature anomalies and patterns of traumatic work-related injuries in workers has also been studied. A review of agricultural workers’ compensation claims showed that the risk of work-related injuries during heatwaves (3 or more consecutive days above the 90th percentile temperature during the warm season) was 6% higher than on non-heatwave days (95% CI 1.03, 1.08) [30]. This was further characterized by Xiang et al. who found both a significant increase in outdoor workers’ injury claims during heatwaves (3 or more consecutive days with daily maximum temperatures ≥ 35 °C) and an increase in the risk of being hit by moving objects (1.097, 95% CI 1.002,1.202) [31].

#### Surgical site infection

Nine articles described the impact of climate change on surgical outcomes and the development of postoperative complications. Some of these studies found that increasing temperatures were associated with increasing incidence of surgical site infections (SSIs) [32–36]. A retrospective cohort of patients who underwent body contouring surgery were analyzed as two groups, warm weather and cold weather based on the average temperature on date of surgery [32]. The authors found a statistically significant increase in the rate of SSIs in the warm weather group compared to the cold weather group (10.29% vs 4.08%; *p* = 0.0071). Patients operated on in warmer months had a 2.693 times higher risk of SSI compared to colder months.

When temperature was also analyzed as a continuous variable, Anthony et al. found that the average temperature during the month in which a patient was hospitalized was a risk factor for SSI and the odds of developing an SSI increased by 2.1% for every 2.8 °C increase in the average monthly temperature [33]. When categorized into temperature increments, the highest temperature group, >32.2 °C, had an increase in the odds of an SSI of 28.9% (95% CI 20.2, 38.3%) compared to temperatures <4.4 °C. Another study looked at the average temperature specifically on the day of surgery and similarly found that increasing temperatures increased the risk of developing an SSI (RR 1.013; 95% CI 1.001,1.025; *p* = 0.03) [34]. This effect was particularly concentrated in clean cases (RR 1.020; 95% CI 1.001,1.039; *p* = 0.03) and temperatures greater than 90th percentile (24.7 °C) showed an increased effect on the risk of SSIs (RR 1.196; 95% CI 1.055, 1.355, *p* = 0.005) [35]. For every 5 °C increase in average temperature, there was a 2% increase in the odds of SSI. They further went on to map the risk of future climate-associated SSI in the United States using two emission scenarios, medium stabilization and worst-case high emissions scenarios. In both scenarios, the Southeastern region of the United States was predicted to have the highest increase in climate-related SSIs. An analysis of data from the German nosocomial infection surveillance system found that the rates of SSIs increased as the mean monthly temperatures increased [36]. Additionally, on logistic regression analysis, there was a correlation between the occurrence of SSI and higher temperatures, and superficial SSIs had a stronger association with increased temperatures than deep SSIs. They further identified that for certain pathogens, there was a particularly strong association: for Acinetobacter species, there was a 6% increase per additional 1 °C, and for Enterobacter species, there was a 4% increase per additional 1 °C.

In contrast, in a single-institution retrospective review, Ramos et al. found that temperature was not significantly associated with incidence of SSI (Pearson correlation coef. −0.035; *p* = 0.791) [37]. Additionally, a retrospective analysis of SSI rates after 15 of the most common surgical procedures at Duke University Medical Center found that the rate of SSI was highest in the summer (defined as July through September), a finding which did not persist when stratified by surgical procedure [38].

#### Postoperative complications

Postoperative outcomes during times of extreme temperatures were also evaluated [39, 40]. In a prospective analysis of elderly patients (age > 60 years) recovering in environments without routine air conditioning, those undergoing surgery when ambient temperatures exceeded 30 °C had higher rates of complications compared to those undergoing surgery in ambient temperatures of 20–30 °C [39]. These patients also had a longer length of stay (13.21 ± 6.44 vs 9.81 ± 3.54 days; *p* = 0.01), although the impact of temperature on complications was not found to be statistically significant on multivariate analysis (coef. 0.091; *p* >0.10). Another prospective study of surgical trauma patients recovering without routine air conditioning found a higher rate of complications in patients who had surgery when the outdoor temperature was greater than 40 °C compared to when it ranged between 20 and 29 °C (54% vs 20%; *p* = 0.009) [40]. On multivariate analysis, peak environmental temperature was the only independent risk factor for predicting perioperative complications (*p* = 0.003), such as myocardial infarction, acute renal failure, and multiple organ dysfunction syndromes. They also found that this effect was modified for patients of lower socioeconomic status, who experienced higher rates of complications if they were operated upon during periods of high ambient temperature.

## Discussion

The impact of healthcare on global warming is becoming increasingly recognized. However, the impact of climate change on surgery has yet to be explored. We conducted a comprehensive review to evaluate the scope and depth of the existing literature regarding the intersection of climate change and general surgery. Our findings can be assessed through two major consequences of climate change: extreme weather events and temperature anomalies. Current evidence suggests that extreme weather events have contributed to an increase in surgical need and exacerbated the burden of surgical diseases due to impaired access to care. Temperature anomalies may increase the surgical burden of common surgical diseases, worsen postoperative outcomes, and increase the risk of injuries. Our scoping review highlighted significant knowledge gaps and limited understanding of the possible implications of climate change on surgical care and its delivery.

The impact extreme weather events on surgical care and surgical service delivery is multifold. The most obvious are the injuries resulting directly from the event (i.e., wildfire burns or soft tissue wounds after a landslide), which may lead to increased surgical demand. Several studies have also described impaired access to surgical care after natural disasters, including closure of surgical centers, crippled road infrastructure, and interruptions in water and energy services. However, the clinical impact of these barriers appears to be less clear. In some cases, the increased difficulty of receiving expeditious surgical care for routine procedures appeared to worsen the severity and acuity of disease at the time of presentation[20, 21] and worsen surgical outcomes [24], while in others, no differences were found [22, 23]. Additionally, the absence of increased surgical volumes after extreme weather events [14] could reflect a true lack of escalation in acute surgical demand; however, it could also be the result of impaired access to surgical care. Our scoping review demonstrated that these complex, inter-related questions have not been well studied in general surgery. On the other hand, interruptions to care for chronic medical conditions, such as hypertension and diabetes, have been more extensively analyzed and found to result in increased morbidity and worsened disease states [41–44]. Despite reports of disrupted water and energy services after extreme weather events, our search did not identify studies specifically examining how climate-related power outages affect operating room function, anesthesia delivery, or surgical equipment. This is an important gap, particularly for low- and middle-income countries where unstable power grids already threaten safe surgery and may worsen with increasing climatic instability. External reports highlight the need for reliable backup systems, including solar-powered solutions, to maintain OR capacity during outages. Even beyond the scope of our review, most of the current literature on surgical care after extreme weather events focuses on the patterns of injuries seen and types of cases performed by surgical teams after natural disasters [45–47]. While these studies are critical in allowing surgeons to anticipate patient needs, a better understanding is needed of how the burden of surgical disease and postoperative outcomes are affected by impaired access to surgical care after natural disasters. This is of particular significance when considering the analysis conducted by Lee et al., which found that surgical procedure rates are not a direct reflection of the true need for surgical care. Should further study reveal a significant impact from these barriers, hospital systems should seek to identify mitigation strategies by increasing the resilience of their operations to accurately address a changing pattern of surgical demand. Furthermore, evidence seems to suggest that the burden of these effects is disproportionately distributed and exacerbates existing disparities [43, 44]. This inequity is particularly stark in disaster-prone or resource-limited regions, where infrastructure fragility and surgical workforce shortages compound the effects of climate-related events. Without targeted mitigation strategies, these disparities may widen, further limiting access to essential surgical care.

There were also a number of indirect impacts highlighted in the literature. The exposure of patients to extreme weather events may potentially worsen subsequent postoperative outcomes [25], however, we again found few publications on this subject. For example, a recent national study also found that hospital exposure to wildfire disasters was linked to longer postoperative stays after lung cancer surgery, suggesting that such events may indirectly delay recovery even without increasing mortality [27]. Within the obstetrics literature, exposure to a natural disaster has been shown to complicate pregnancies and increase rates of caesarean section and abnormal labor [50–52]. Additionally, the aftermath of the extreme weather events can also result in unforeseen consequences, such as the mass chemical burn casualty due to flooding of a storehouse during the Matsa typhoon [17]. Unfortunately, these indirect outcomes tend to be difficult to study and even more difficult to clearly attribute to effects of climate change.

In contrast, deviations in temperature have been more rigorously analyzed. Extreme heat and heatwaves, which are increasingly more common with climate change [51], were found to increase the burden of surgical disease. Our review identified three studies, which showed a positive correlation between incidence and severity of two common general surgery diseases with increasing ambient temperatures. Heatwaves were also correlated with increases in the rates of work-related traumatic injuries [28, 29]. While difficult to prove, it is speculated that increases in core temperature and dehydration can result in fatigue, vigilance decrement and loss of concentration, leaving workers more prone to injury [29]. These findings are supported by a systematic review of 20 studies by Bonafede et al., which confirmed this relationship between extreme temperature and work-related injuries [52]. Outside of general surgery, there have been studies demonstrating increased rates of renal colic [53], acute otitis externa [54], and optic disk hemorrhage [55] associated with higher average temperatures. This area in particular warrants further study, as there appears to be a consistent pattern throughout surgery of increased surgical burden associated with rising temperatures. The validity of the current literature can be readily assessed by repeating the aforementioned analyses within different geographic regions and in different patient populations. Other common surgical diseases which may be impacted by climate change should also be identified through additional research.

Extreme temperatures were also found to worsen postoperative outcomes—most notably SSIs. The impact of temperature anomalies on the rate and risk of SSIs has been the most extensively evaluated in the literature, with conflicting results. The significant variability in the analytic methods likely contributes to the differences in conclusions. The first difference was in how temperature anomalies were defined throughout the studies. Some authors grouped patients into cohorts based on relative temperature at the time of surgery (i.e., warm seasons vs cold seasons) while others identified anomalies in the average daily, weekly, or monthly temperatures during the perioperative period and correlated these with rates of SSIs during the same period. Additionally, spatial boundaries were also delineated differently, such that a given patient may or may not have truly experienced the temperature anomalies to which they were assigned. Regardless of these differences, the general conclusion of the studies within this scoping review as well as those outside the scope of this project [56–58] is that elevated ambient temperatures appear to be associated with a higher risk of SSIs.

### Limitations

Inherent to the design of scoping reviews, limitations including author bias, article identification, article selection, and article availability must be acknowledged. The definition of climate change employed in the context of this review is a key limitation. While the definition of climate change as “long-term shifts in weather patterns” is widely accepted, a more specific interpretation of what this constitutes was needed for this review. We included only certain natural disasters widely thought, but not proven, to be a consequence of climate change, and therefore, the inclusion or exclusion of a given article hinges on the validity of this assumption. The search terms used in the library search strategy—specifically, the omission of “crime” as a term—is a limitation. This may have inadvertently excluded studies examining the relationship between climate change, violent crime, and subsequent surgical interventions. Future research could benefit from an expanded search strategy to include terms related to violent crime to ensure a more comprehensive understanding of the indirect effects of climate change on surgical care. The variability among institutions regarding which surgical specialty manages a given surgical condition prevents the establishment of a universally accepted definition of general surgery. Non-English articles were not considered, nor were gray literature or limited cases series which could result in a failure to adequately identify and include international data, signals from early observational work, or findings from under-resourced research groups. Though addressed individually throughout the review, the retrospective and observational designs featured in most included articles can at best suggest correlations between climate change and outcomes of interest. In lieu of prospective studies which are often not feasible in climate change literature, causative relationships cannot be assessed. Finally, the classification and interpretation of articles is intrinsically subjective and liable to influence from bias.

## Conclusion

Despite widespread recognition of the detrimental impacts of climate change on human health, the specific effects on surgical disease are not well known. This review found the literature in the field of general surgery to be limited and superficial. Current evidence seems to suggest that climate change, through extreme weather events and temperature anomalies, may result in an increase in surgical need, exacerbate the burden of surgical diseases due to impaired access to care, and worsen postoperative outcomes. Further research is needed within this space to improve our understanding of the consequences to the health of our patients.

## Data Availability

The data used in this study are not publicly available but can be provided upon reasonable request.
